# An Atypical Presentation of Pancreatic Pseudocyst Masquerading as Solid Pseudopapillary Neoplasm of Pancreas

**DOI:** 10.7759/cureus.9883

**Published:** 2020-08-19

**Authors:** Satyaprakash Ray Choudhury, Sumit Mohanty, Debahuti Mohapatra, Nibedita Sahoo, Adya Panda

**Affiliations:** 1 Surgical Gastroenterology, Siksha O Anusandhan University Institute of Medical Sciences and SUM Hospital, Bhubaneswar, IND; 2 Pathology, Siksha O Anusandhan University Institute of Medical Sciences and SUM Hospital, Bhubaneswar, IND; 3 Radiology, Siksha O Anusandhan University Institute of Medical Sciences and SUM Hospital, Bhubaneswar, IND

**Keywords:** pancreatic pseudocyst, solid pseudopapillary neoplasm, cystic neoplasm of the pancreas

## Abstract

Pancreatic pseudocysts are the most common cystic lesions of the pancreas, and often present as a consequence of acute or chronic pancreatitis. On the other hand, cystic neoplasms of the pancreas are rare, but pose a significant diagnostic challenge. The differentiation between these entities often relies on the clinical features and characteristic radiological evidence. However, the diagnostic dilemma persists, leading to misdiagnosis and inappropriate treatment. We present a case of pancreatic pseudocyst in a 49-year-old male, which clinically and radiologically mimicked solid pseudopapillary neoplasm, a rare type of cystic neoplasm of the pancreas.

## Introduction

Pancreatic pseudocyst is the most common cystic lesion of the pancreas accounting for 80% of cases and often presents as a sequel of acute or chronic pancreatitis [1]. In contrast, most of the cystic neoplasms present with vague upper abdominal symptoms or maybe incidental findings only [2]. The solid pseudopapillary neoplasm (SPN) represents a rare type of cystic neoplasms of the pancreas with low malignant potential [3]. The preoperative diagnosis of cystic lesions of the pancreas often entails an array of investigations, but in many cases, the definitive diagnosis remains uncertain till final histopathology. We present a rare case of pancreatic pseudocyst that strongly mimicked SPN on preoperative diagnostic tools, and was managed with pancreatoduodenectomy. 

## Case presentation

A 49-year-old male without any comorbidities presented with mild upper abdominal pain, anorexia, and occasional vomiting for two months. He had a history of chronic alcohol abuse for 12 years and smoking for 20 years. He did not have a pain abdomen before this event. He denied any history of abdominal trauma. His physical examinations were unremarkable.

**Figure 1:**
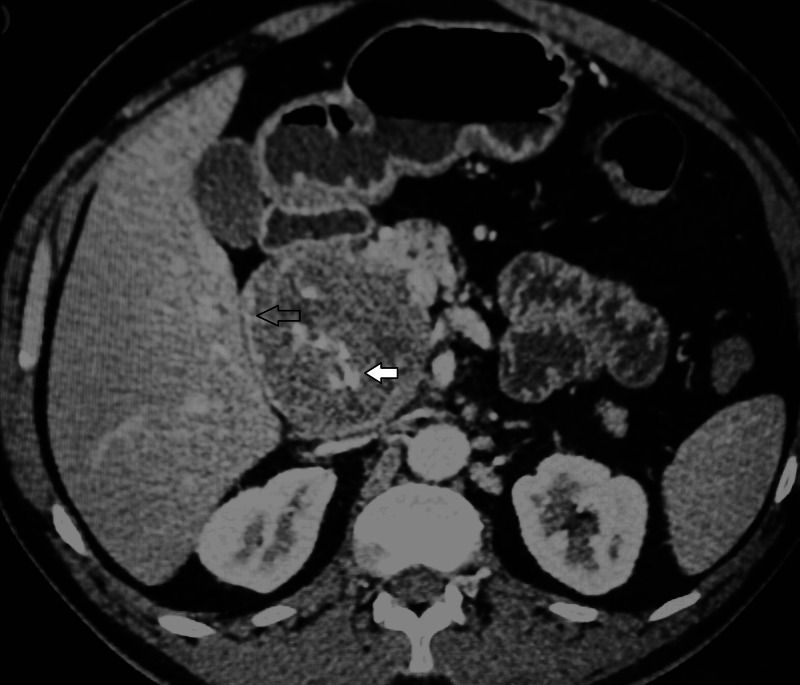
CECT scan of the abdomen, axial view, showing well-capsulated solid lesion in head and uncinate process of pancreas, with capsular enhancement (marked with blank arrow) and central scattered calcification (marked with solid arrow) CECT: contrast-enhanced computed tomography

**Figure 2:**
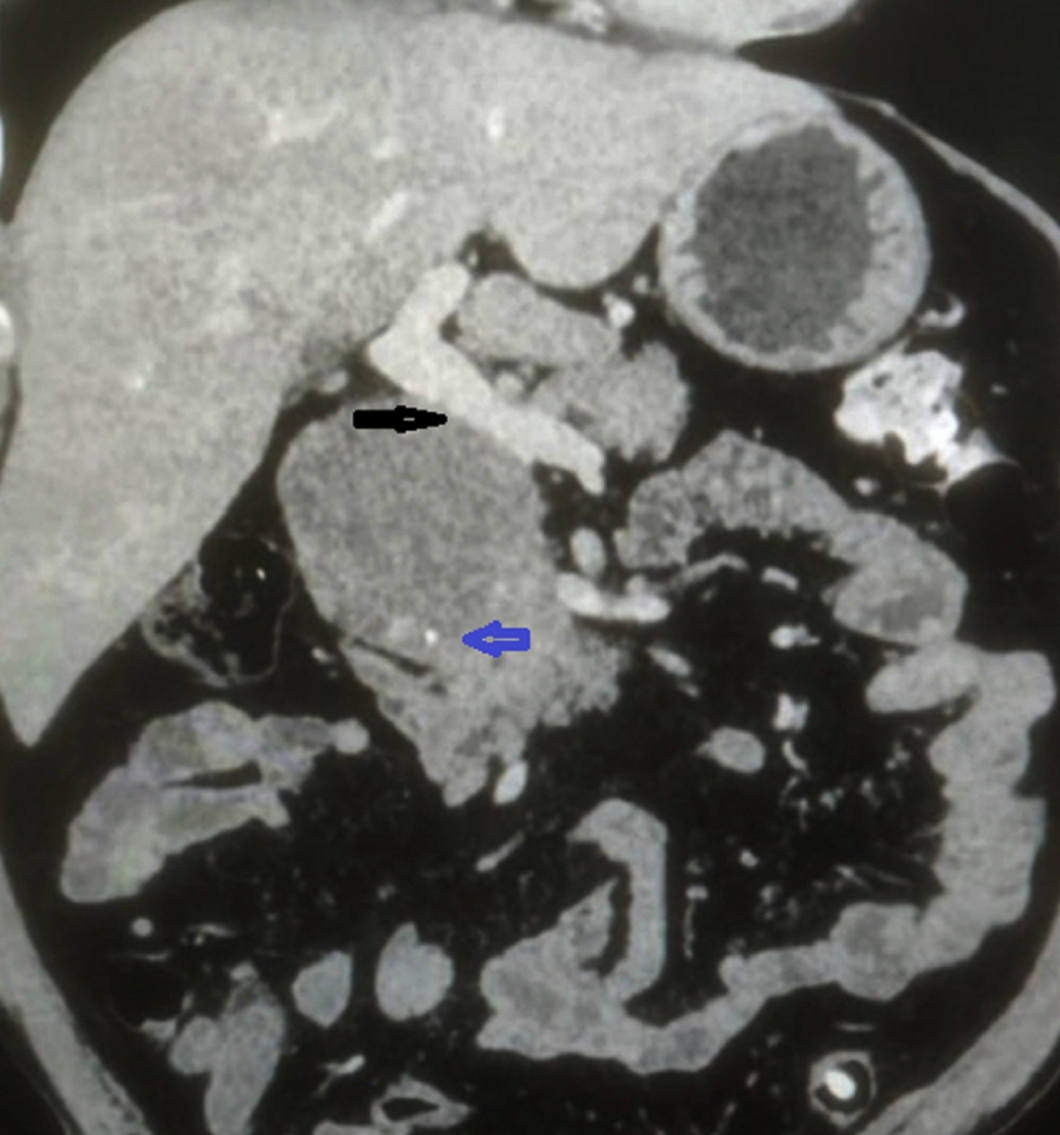
CECT scan of the abdomen, coronal view, showing the lesion closely abutting the portal vein with discontinuity of capsule (marked by black arrow), and peripheral calcification (marked with blue arrow) CECT: contrast-enhanced computed tomography

Contrast-enhanced computed tomography (CECT) showed a well-defined solid, round, hypo to isodense lesion of size 7 × 7 × 6.5 cm arising from the head and uncinate process of the pancreas with a well-defined capsule, which showed enhancement in arterial and portal phase. There were multiple scattered foci of central and peripheral calcifications noted inside the lesion. It was abutting and compressing the portal and superior mesenteric vein confluence with the focal discontinuity of capsule (Figures 1, 2). The rest of the pancreatic parenchyma showed normal enhancement, preserved lobulation, and undilated duct.

 

**Figure 3:**
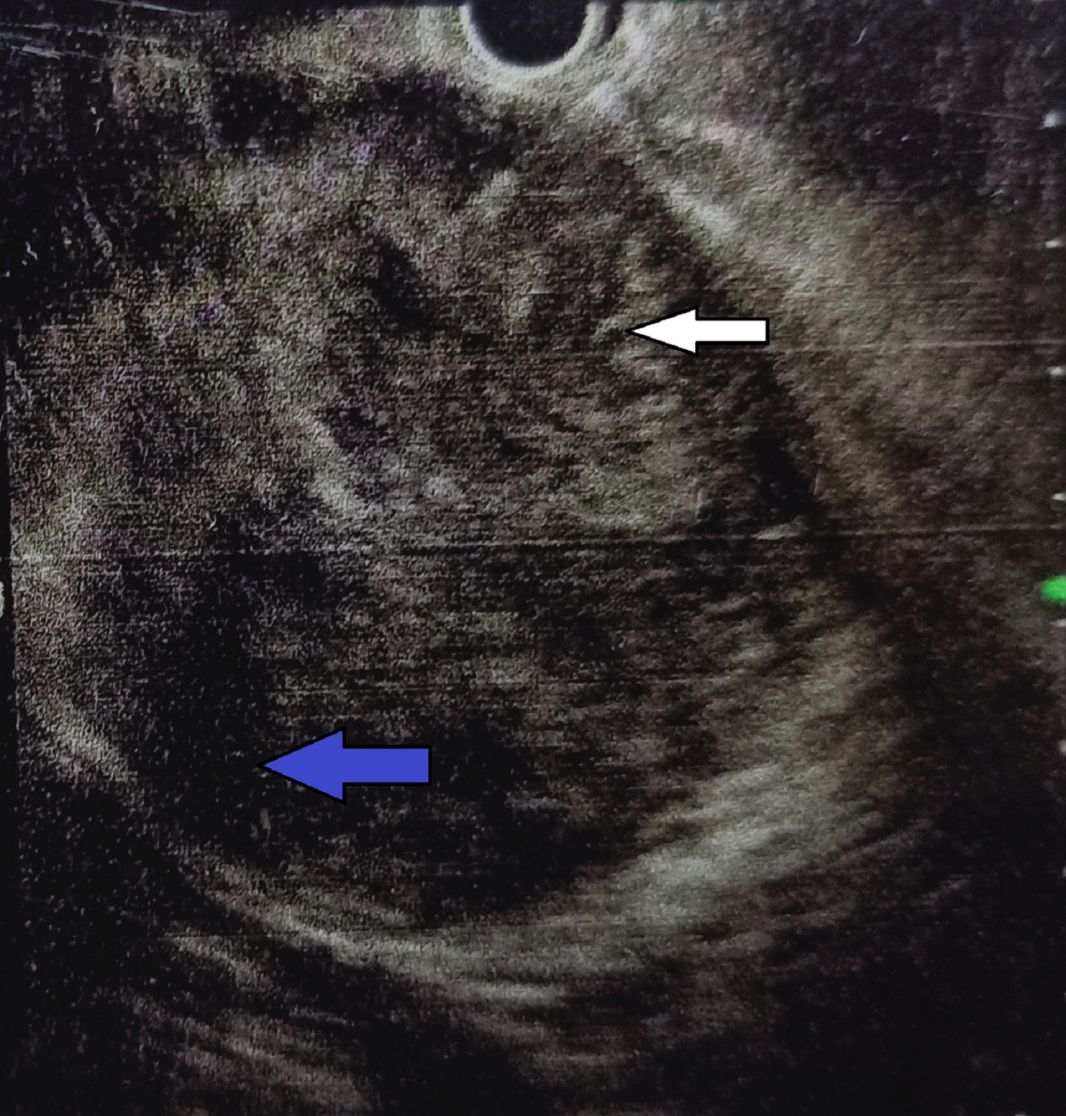
EUS picture showing solid component (marked with white arrow) with cystic degeneration (marked with blue arrow) EUS: endoscopic ultrasound

**Figure 4:**
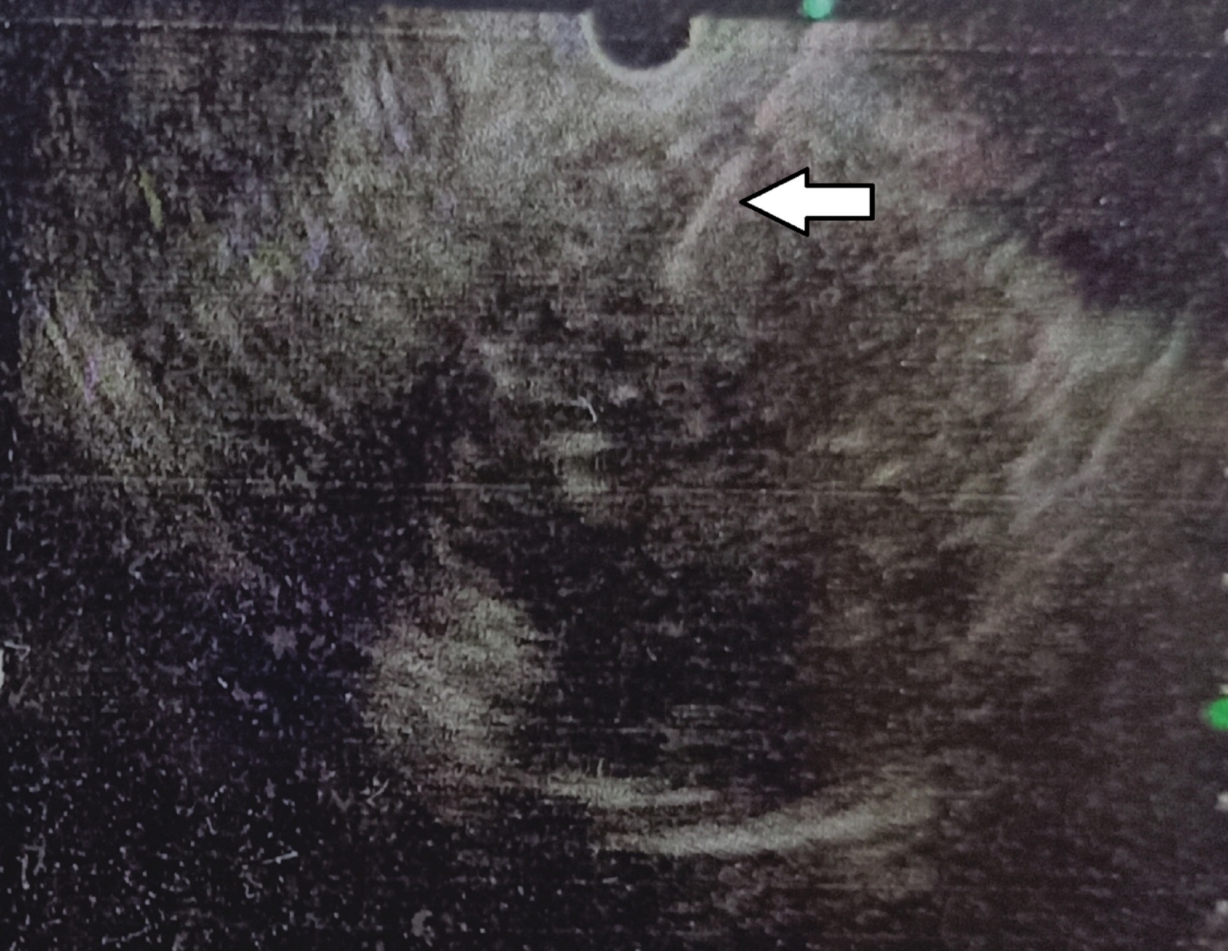
EUS-guided FNAC taken from solid component (marked with arrow) EUS: endoscopic ultrasound, FNAC: fine needle aspiration cytology

In suspicion of cystic neoplasm of the pancreas, endoscopic ultrasound (EUS) was performed, which revealed well-defined, hypo to isoechoic lesion with multiple small foci of calcification and cystic degeneration (Figures 3, 4). EUS-guided fine needle aspiration cytology (FNAC) from solid component showed only benign columnar epithelial cells. The aspiration from the cystic area showed thick material, but further analysis of the cyst fluid was unfortunately unavailable. The CA 19-9 and serum amylase levels were 24 U/mL and 47 U/L, respectively.

Because of radiological findings highly suggestive of cystic neoplasm of the pancreas with possible malignant transformation, surgical resection was planned. He underwent pylorus resecting pancreatoduodenectomy. There was a large, well-capsulated cystic mass arising from the head of the pancreas and closely abutting the portal vein, which could be separated without damaging the capsule. The postoperative period was uneventful, and he was discharged on postoperative day 7.

**Figure 5:**
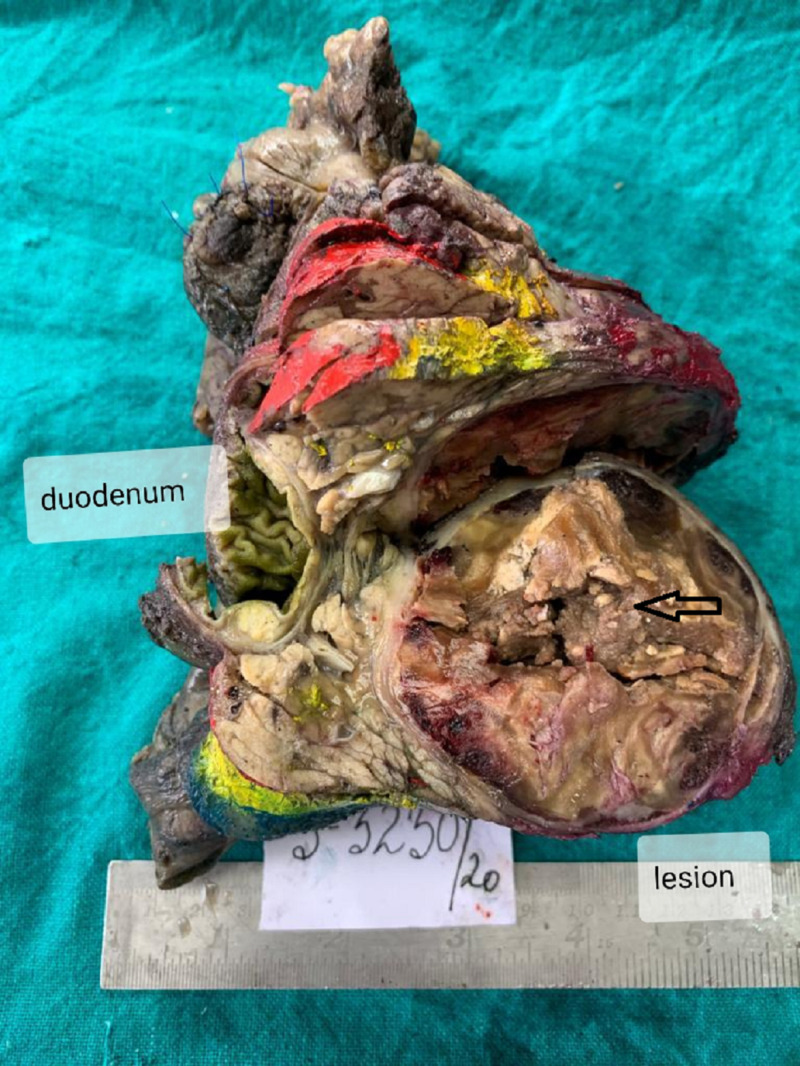
Gross examination of specimen showing well-encapsulated necrohemorrhagic cyst with areas of calcification (marked with arrow)

**Figure 6:**
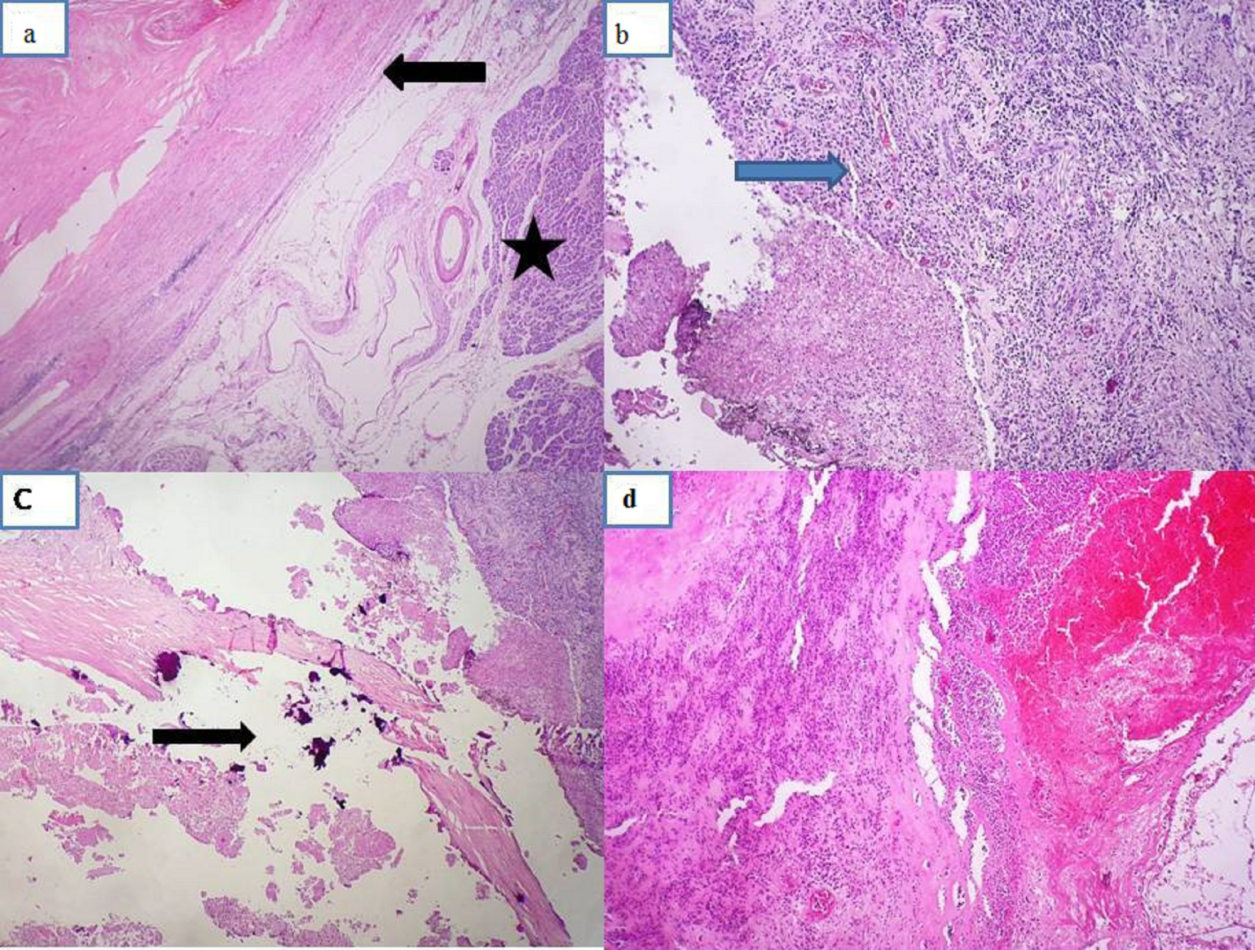
(a) Histopathological examination showing cystic structure with hyalinized fibrocollagenous wall (arrow) and adjoining pancreatic tissue (marked with asterisk) (H&E ×40). (b) Cyst wall showing unhealthy granulation tissue and lacks epithelial lining(marked with blue arrow) (H&E ×100). (c) Areas of calcification (marked with arrow). (d) Cyst containing acute inflammatory exudates, necrotic and hemorrhagic material (H&E ×40).

The gross examination of the specimen showed a thick-walled capsulated necrohemorrhagic cystic lesion with areas of calcification (Figure 5). Histopathological examination revealed extensive areas of necrosis, hemorrhage, and dystopic calcifications along with dense acute inflammatory infiltrate. The wall showed unhealthy granulation tissue with chronic inflammatory cells forming lymphoid aggregates in places. There were no epithelial lining or malignant cells, consistent with pancreatic pseudocyst (Figure 6).

## Discussion

Cystic neoplasms of the pancreas are rare and comprise less than 10% of all pancreatic malignancies [4]. Although the modern diagnostic modalities like thin slice CECT, MRI with magnetic resonance cholangiopancreatography, and EUS differentiate between the cystic lesions of the pancreas with fair accuracy, there are several reports where they have failed to correctly identify the lesion, and final diagnosis has relied on the histopathology. In one study, the misdiagnosis occurred in 44% of cases, and nearly 8% of incidental non-neoplastic cysts were removed [2]. In our case, the CT findings and the EUS features strongly suggested SPN of the pancreas.

SPN accounts for 1% of all pancreatic neoplasms with high female predominance [5]. Although it is most prevalent in young women of the second decade of life, approximately 17% of cases are males with a higher mean age of presentation [6]. SPN is composed of poorly cohesive epithelial cells arranged in solid and pseudopapillary structures [7]. These frequently undergo hemorrhagic cystic degeneration to give the characteristic solid and cystic appearance on radiological examinations, especially on EUS [8]. The ratio of solid to cystic components tends to be higher in males than females, showing that males have a slower rate of degeneration than females [8]. Calcification in SPN has been observed in 30% of cases [6]. Several patterns of calcification, including focal nodular, amorphous or scattered, incomplete or complete rim calcifications, have been observed in SPN, though no calcification pattern is significantly associated with malignant transformation [9]. Approximately 15% to 20% of SPN present with invasion or metastasis at presentation [10,11]. The size of more than 6 cm, vascular invasion, focal discontinuity of capsule, and pancreatic tail location are found to be significantly associated with malignancy in SPN [9]. 

In our case, a well-encapsulated solid lesion with a typical calcification pattern in CECT and solid component with cystic degeneration in EUS strongly suggested SPN.

Pancreatic pseudocyst may simulate clinically and radiologically with other cystic neoplasms of the pancreas. Serous cystic neoplasm (SCN) mostly affects females of the sixth or seventh decade of life and favors the body and tail of the pancreas [12]. Central stellate scar, sunburst calcification, lobulated appearance, septations, and micro or macrocysts are characteristic features of SCN [13]. Mucinous cystic neoplasm (MCN), the most common cystic neoplasm of the pancreas, is exclusively seen in females [14]. Although most of the cases present as unilocular cyst at the tail region, it can involve any part of the pancreas. Unilocular or multilocular cysts, enhancing septations, and peripheral calcification on CECT suggest MCN, though calcification is seen in 10%-25% of the cases [15]. The more typical findings on EUS are multiple macrocystic locules giving a ‘cyst in cyst’ appearance, dividing septations, and thick mucoid content [16]. These features were absent in our case. Intraductal papillary mucinous neoplasm (IPMN) is the mucin secreting tumor of the main or branched pancreatic duct, which commonly affects males [14]. Pancreatic pseudocyst located at the head and uncinate process of the pancreas may mimic IPMN, especially when no history of pancreatitis is evident. The dilated main pancreatic duct, punctuate or coarse calcifications, and mural nodules are characteristic findings in main duct IPMN [17]. Branched duct IPMN gives a 'bunch of grapes' appearance on EUS, as the mucin-filled dilated branched ducts arrange themselves in pleomorphic pattern [16]. The communication between the cyst and main pancreatic duct differentiates IPMN from MCN. In our case, the main pancreatic duct was normal, and there was no communication of lesion with the pancreatic duct. Moreover, it was a single encapsulated lesion having solid and cystic components. Hence, IPMN was less considered.

Pancreatic pseudocyst usually occurs as a sequela to acute pancreatitis, chronic pancreatitis, or pancreatic trauma [18]. However, pancreatic pseudocyst can occur as an incidental radiological finding only. On imaging, they are unilocular with enhancing cyst walls without mural nodules or septations. Only in 10% to 20% of cases, the pseudocysts appear multilocular [19,20]. The debris inside the pseudocyst, if present, is detected on EUS as hyperechoic material and may mimic with thick mucus in MCN. However, the change in decubitus of the patient confirms the presence of floating debris. Besides, imaging features of acute pancreatitis like the bulky pancreas, peripancreatic fat stranding, interstitial edema, or features of chronic pancreatitis like the atrophic pancreas, dilated pancreatic duct, and intraductal and intraparenchymal calcifications often accompany pancreatic pseudocyst [18]. In contrast to cystic neoplasm of the pancreas, calcification is extremely rare in pancreatic pseudocyst. Calcifications in pseudocyst, when present, are because of long-standing disease and often accompany chronic pancreatitis. Moreover, the calcification in pseudocyst is peripherally located or eggshell type but is rare [18].

In our patient, the clinical and radiological features were highly suspicious of SPN. Furthermore, the lesion was more than 7 cm and was abutting portal vein with an area of suspected focal discontinuity of capsule, which suggested malignant transformation. Hence, the decision for pancreatoduodenectomy was taken, which is a too morbid procedure for pancreatic pseudocyst. We lacked cyst fluid analysis in our preoperative diagnostic protocol, which might have helped towards the right diagnosis. The aspirated material in both pseudocyst and SPN yields bloody and necrotic debris, but high cyst fluid amylase level along with cyst wall biopsy might have supported the diagnosis of a pseudocyst. 

## Conclusions

Pancreatic pseudocyst may resemble strongly to the cystic neoplasm of the pancreas, especially when there is no history suggestive of pancreatitis. Long-standing pancreatic pseudocyst may calcify and mimic SPN. This case represents a unique challenge in the diagnosis of pancreatic pseudocyst and differentiating it from other cystic neoplasms. We emphasize that the preoperative diagnostic protocol should include EUS-guided cyst fluid analysis along with radiological investigations when an atypical diagnosis is made or in case of a diagnostic dilemma, thereby avoiding unnecessary morbid procedures.

## References

[1] Brugge WR (2015). Diagnosis and management of cystic lesions of the pancreas. J Gastrointest Oncol.

[2] Fernández-del Castillo C, Targarona J, Thayer SP, Rattner DW, Brugge WR, Warshaw AL (2003). Incidental pancreatic cysts: clinicopathologic characteristics and comparison with symptomatic patients. Arch Surg.

[3] Yao J, Song H (2020). A review of clinicopathological characteristics and treatment of solid pseudopapillary tumor of the pancreas with 2450 cases in Chinese population. Biomed Res Int.

[4] Scheiman JM, Hwang JH, Moayyedi P (2015). American Gastroenterological Association technical review on the diagnosis and management of asymptomatic neoplastic pancreatic cysts. Gastroenterology.

[5] Martin RC, Klimstra DS, Brennan MF, Conlon KC (2002). Solid-pseudopapillary tumor of the pancreas: a surgical enigma?. Ann Surg Oncol.

[6] Park MJ, Lee JH, Kim JK (2014). Multidetector CT imaging features of solid pseudopapillary tumours of the pancreas in male patients: distinctive imaging features with female patients. Br J Radiol.

[7] Tang LH, Aydin H, Brennan MF, Klimstra DS (2005). Clinically aggressive solid pseudopapillary tumors of the pancreas: a report of two cases with components of undifferentiated carcinoma and a comparative clinicopathologic analysis of 34 conventional cases. Am J Surg Pathol.

[8] Takahashi Y, Hiraoka N, Onozato K, Shibata T, Kosuge T, Nimura Y, Kanai Y, Hirohashi S (2006). Solid-pseudopapillary neoplasms of the pancreas in men and women: do they differ?. Virchows Arch.

[9] Yin Q, Wang M, Wang C (2012). Differentiation between benign and malignant solid pseudopapillary tumor of the pancreas by MDCT. Eur J Radiol.

[10] Kim MJ, Choi DW, Choi SH, Heo JS, Sung JY (2014). Surgical treatment of solid pseudopapillary neoplasms of the pancreas and risk factors for malignancy. Br J Surg.

[11] Kang CM, Choi SH, Kim SC, Lee WJ, Choi DW, Kim SW, Korean Pancreatic Surgery Club (2014). Predicting recurrence of pancreatic solid pseudopapillary tumors after surgical resection: a multicenter analysis in Korea. Ann Surg.

[12] Dietrich CF, Dong Y, Jenssen C (2017). Serous pancreatic neoplasia, data and review. World J Gastroenterol.

[13] Ishigami K, Nishie A, Asayama Y (2014). Imaging pitfalls of pancreatic serous cystic neoplasm and its potential mimickers. World J Radiol.

[14] Crippa S, Fernández-Del Castillo C, Salvia R (2010). Mucin-producing neoplasms of the pancreas: an analysis of distinguishing clinical and epidemiologic characteristics. Clin Gastroenterol Hepatol.

[15] Hutchins GF, Draganov PV (2009). Cystic neoplasms of the pancreas: a diagnostic challenge. World J Gastroenterol.

[16] Tanaka M, Chari S, Adsay V (2006). International consensus guidelines for management of intraductal papillary mucinous neoplasms and mucinous cystic neoplasms of the pancreas. Pancreatology.

[17] Bollen TL, Wessels FJ (2018). Radiological workup of cystic neoplasms of the pancreas. Visc Med.

[18] Javadi S, Menias CO, Korivi BR, Shaaban AM, Patnana M, Alhalabi K, Elsayes KM (2017). Pancreatic calcifications and calcified pancreatic masses: pattern recognition approach on CT. AJR Am J Roentgenol.

[19] Gonzalez Obeso E, Murphy E, Brugge W, Deshpande V (2009). Pseudocyst of the pancreas: the role of cytology and special stains for mucin. Cancer.

[20] Kubo H, Nakamura K, Itaba S (2009). Differential diagnosis of cystic tumors of the pancreas by endoscopic ultrasonography. Endoscopy.

